# Aggressive primary mediastinal synovial sarcoma resulting in catastrophic airway compromise and fatal hemoptysis

**DOI:** 10.1016/j.radcr.2026.09.032

**Published:** 2026-10-03

**Authors:** Steven Liu, Ravi Malpani, Sophia Lubrin, Vatche Israbian

**Affiliations:** a Department of Internal Medicine, Parkview Health, 11109 Parkview Plaza Drive, Fort Wayne, IN 46845, USA; b Department of Critical Care, Parkview Health, 11109 Parkview Plaza Drive, Fort Wayne, IN 46845, USA; c Department of Pulmonology, Parkview Health, 11109 Parkview Plaza Drive, Fort Wayne, IN 46845, USA

**Keywords:** Synovial sarcoma, Mediastinal mass, Airway obstruction, Cardiac tamponade, Stents, Disease progression

## Abstract

Primary mediastinal synovial sarcoma is a rare and aggressive malignancy that may present with nonspecific cardiopulmonary symptoms and be occult on chest radiography. A 45-year-old woman presented with progressive cough, fatigue, and chest discomfort despite an initially unremarkable chest radiograph. Six weeks later, worsening dyspnea, orthopnea, and dysphagia prompted repeat imaging. Chest radiography demonstrated tracheal deviation, and computed tomography angiography revealed an 11.3 cm infiltrative mediastinal mass compressing the central airways, pulmonary vasculature, and left atrium, with an associated pericardial effusion. Endobronchial ultrasound-guided biopsy confirmed biphasic synovial sarcoma with an SS18 rearrangement. The patient subsequently developed cardiac tamponade requiring a pericardial window. The tumor was unresectable because of extensive cardiopulmonary involvement. Despite silicone Y-stent placement and planned chemotherapy, she developed airway obstruction, massive hemoptysis, and fatal respiratory failure. This case demonstrates the rapid progression and life-threatening mechanical complications of primary mediastinal synovial sarcoma. Persistent or progressive cardiopulmonary symptoms should prompt cross-sectional imaging when initial chest radiography is unrevealing.

## Introduction

Primary mediastinal synovial sarcoma is a rare and aggressive soft tissue malignancy. Primary mediastinal sarcomas account for approximately 1–2% of all soft tissue sarcomas and comprise less than 10% of primary mediastinal tumors [1,2]. More broadly, thoracic soft tissue sarcomas represent only a very small fraction of thoracic malignancies, with reported prevalence as low as 0.01% of all thoracic cancers [3]. Synovial sarcoma most commonly arises in the extremities near large joints of adolescents and young adults, whereas primary mediastinal involvement is exceptionally uncommon [[1], [2], [3], [4]].

Patients typically present with nonspecific symptoms such as cough, dyspnea, chest discomfort, fatigue, orthopnea, or dysphagia, which may initially resemble more common benign cardiopulmonary conditions and delay recognition [2,3,5]. Because the mediastinum can accommodate substantial tumor growth before symptom onset, diagnosis is often delayed until significant mass effect develops. This may result in complications including malignant central airway obstruction, pulmonary artery compression, pericardial effusion, and cardiac tamponade [1,2,6].

Despite aggressive multidisciplinary management—including thoracic surgery, interventional pulmonology, and systemic chemotherapy—rapid disease progression may outpace available therapeutic interventions [2,5,7].

We present a case of primary mediastinal biphasic synovial sarcoma distinguished by exceptionally rapid clinical and radiologic progression over a six-week interval. Despite an initially unremarkable chest radiograph, the patient subsequently developed an extensive infiltrative mediastinal mass causing cardiac tamponade, malignant central airway obstruction, and ultimately fatal hemoptysis suspected to result from vascular invasion. The novelty of this case lies in the documented short-interval progression from radiographically occult disease to unresectable cardiopulmonary involvement and multiple life-threatening mechanical complications. This case highlights the limitations of chest radiography in excluding early mediastinal disease and emphasizes the importance of timely cross-sectional imaging when cardiopulmonary symptoms persist or progress.

## Case Presentation

A 45-year-old female and lifelong nonsmoker with a history of hypertension and insomnia developed a progressive nonproductive cough, fatigue, and chest discomfort. She initially presented to the emergency department at symptom onset, where chest radiography demonstrated no significant cardiopulmonary or mediastinal abnormalities, and she was discharged with supportive care (Fig. 1).

**Fig. 1:**
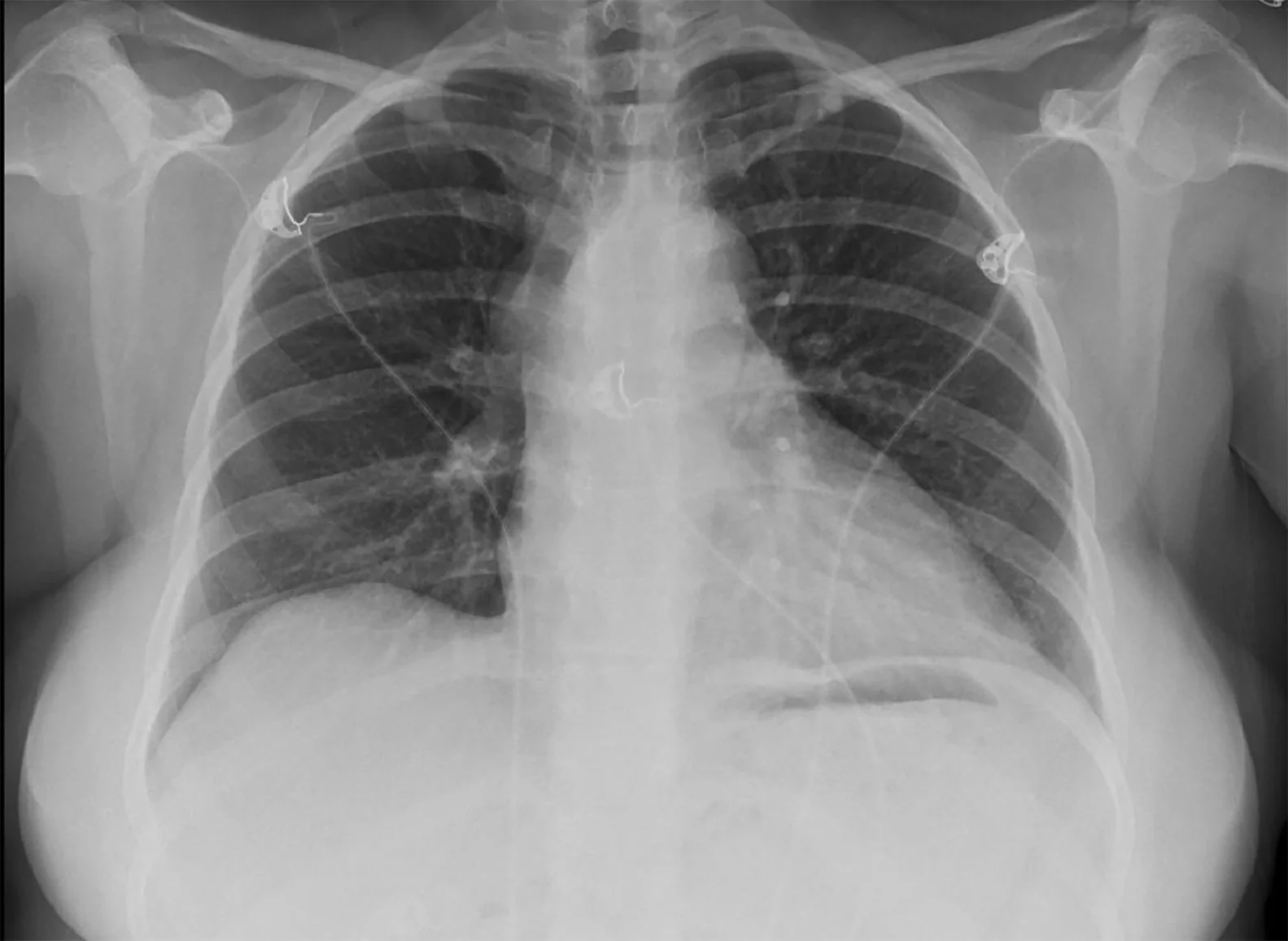
Initial chest X-ray at symptom onset was unremarkable, with no significant pulmonary or cardiomediastinal abnormalities.

Over the following six weeks, her symptoms worsened, progressing to dyspnea, orthopnea, dysphagia, and chest pressure. At that time, repeat chest radiography demonstrated rightward tracheal deviation (Fig. 2), prompting further evaluation with computed tomography angiography (CTA). CTA subsequently revealed a large infiltrative mediastinal mass (8.2 × 5.1 × 11.3 cm) compressing the bilateral main pulmonary arteries, trachea, carina, left atrium, and superior left pulmonary veins, with invasion of the posterior pericardial extension and an associated moderate pericardial effusion (Fig. 3). The extensive airway, vascular, and pericardial involvement was concerning for locally advanced disease.

**Fig. 2:**
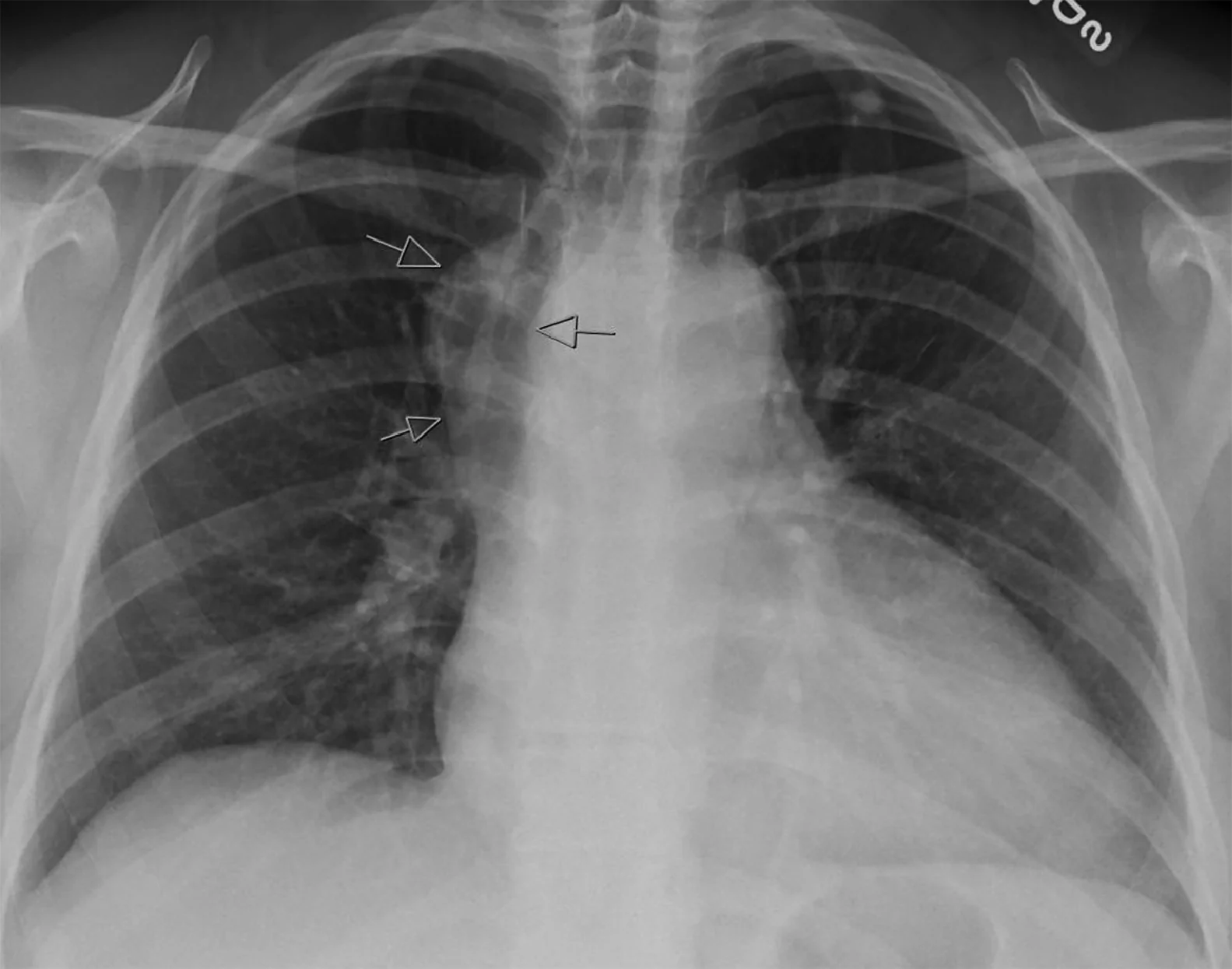
Repeat chest X-ray six weeks later, during worsening symptoms, demonstrated a mass effect with rightward deviation of the trachea at the level of the aortic arch.

**Fig. 3:**
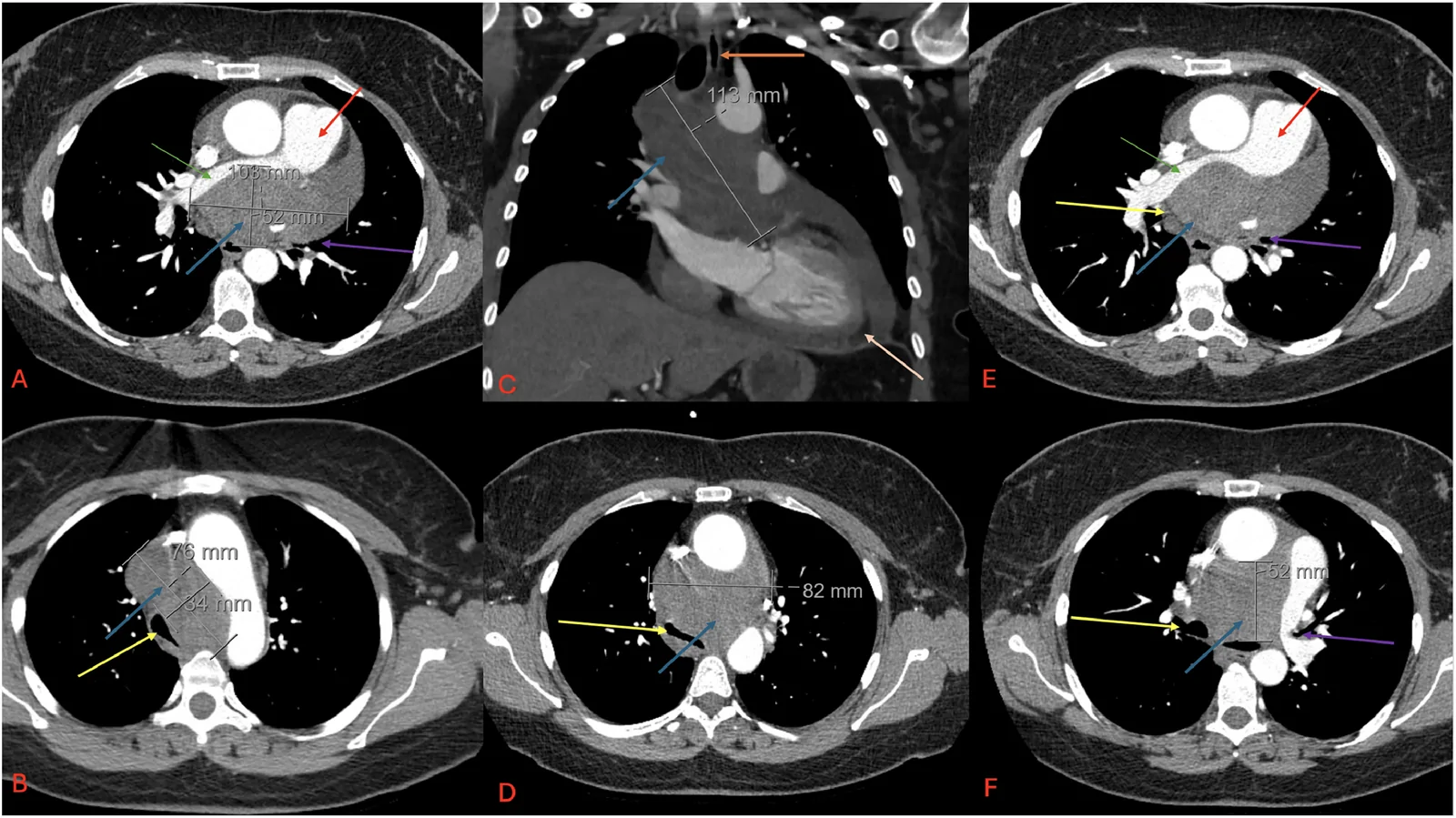
Follow-up CT angiography of the chest. (A) Posterior pericardial component; (B) anterior pericardial component; (C) craniocaudal height measurement of the mediastinal mass; (D) length measurement of the mass; (E) compression of the pulmonary vasculature and left atrium; and (F) transverse width measurement of the mass. The red arrow identifies the main pulmonary artery/right ventricular outflow tract, the teal arrow identifies the mediastinal mass, the green arrow identifies the compressed right pulmonary artery, the yellow arrow identifies the compressed right main bronchus, and the purple arrow identifies the compressed left main bronchus.

One week after the initial chest CTA, she underwent endobronchial ultrasound (EBUS)-guided bronchoscopy, which demonstrated extensive tracheobronchial distortion and extrinsic compression (Fig. 4). Additionally, EBUS demonstrated lymph node involvement at stations 10L, 7, 4R, and 4L. Pathology confirmed primary mediastinal biphasic synovial sarcoma with positive staining for CD99 and TLE1 and an SS18 gene rearrangement by fluorescence in situ hybridization (FISH) (Fig. 5).

**Fig. 4:**
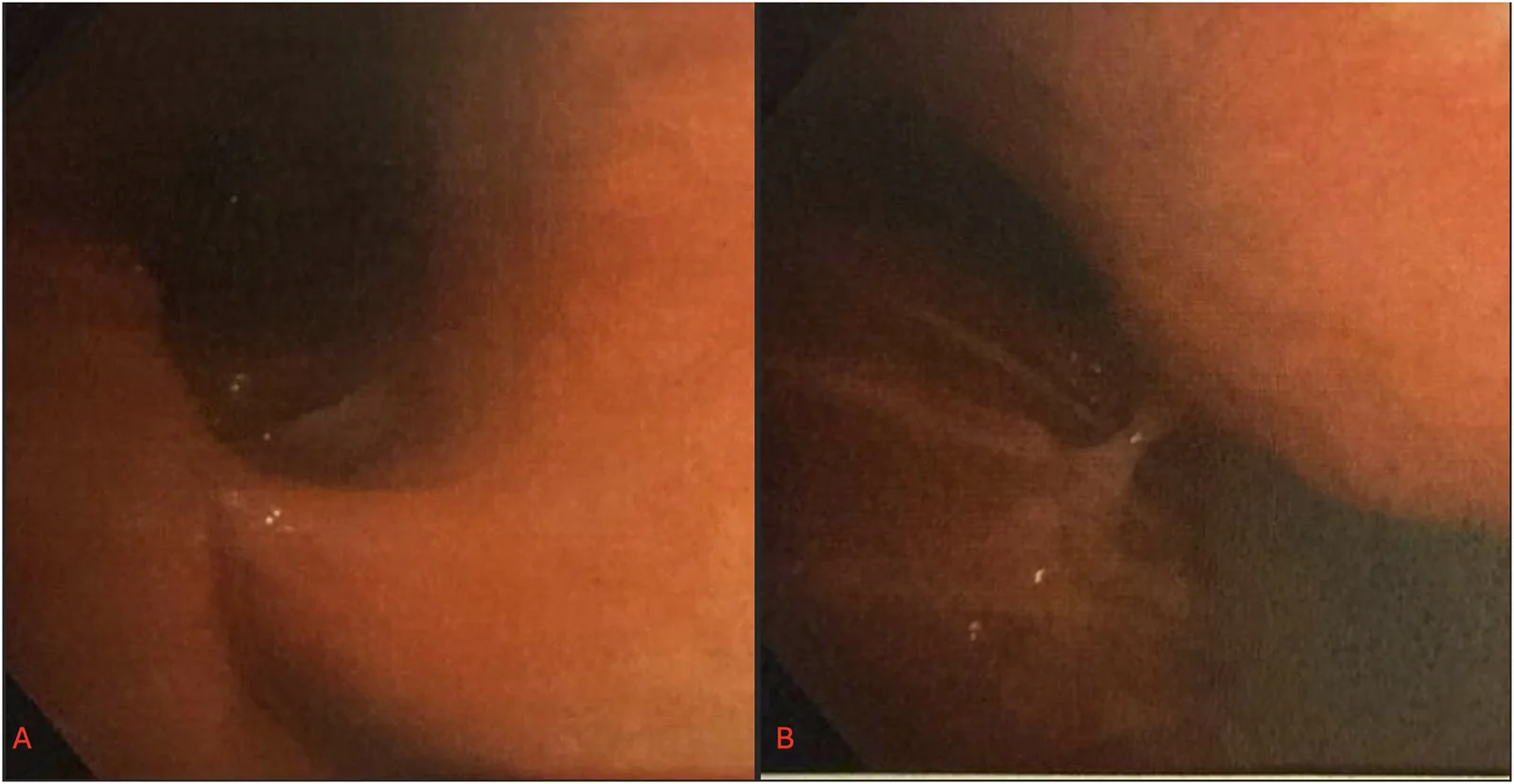
Bronchoscopy images. (A) The left lower lobe bronchus is markedly distorted, narrowed, and compressed, with no endobronchial tumor identified. (B) There is extensive distortion of the trachea, along with erythema and inflammatory changes of the distal trachea. An infiltrative process is noted at the level of the carina, primarily involving the anterior tracheal wall and left mainstem bronchus.

**Fig. 5:**
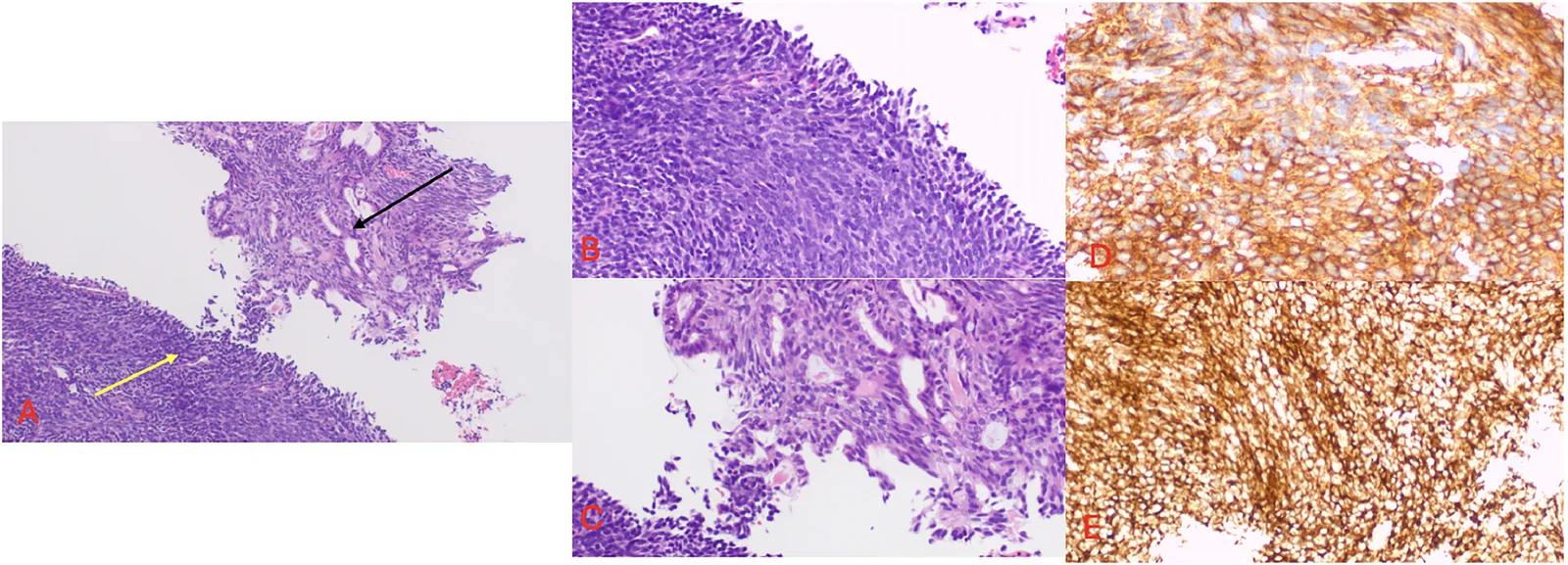
Lymph node pathology: A) Low-power image demonstrating a biphasic neoplasm composed of glandular structures (black arrow) and spindle cells (yellow arrow). (B) High-power image of the glandular component. (C) High-power image of the spindle-cell component. (D) BCL2 immunohistochemistry demonstrating diffuse membranous staining of the tumor cells. (E) CD99 immunohistochemistry demonstrating membranous staining of the tumor cells.

Five days after biopsy, an outpatient transthoracic echocardiogram performed as part of the diagnostic workup demonstrated a large pericardial effusion (5.3 cm) with right heart compression and findings consistent with cardiac tamponade (Fig. 6). She was directed to the emergency department and subsequently underwent an emergent pericardial window. Pericardial cytology was obtained and was negative for malignancy. Her symptoms transiently improved following the procedure, and staging imaging demonstrated no distant metastases.

**Fig. 6:**
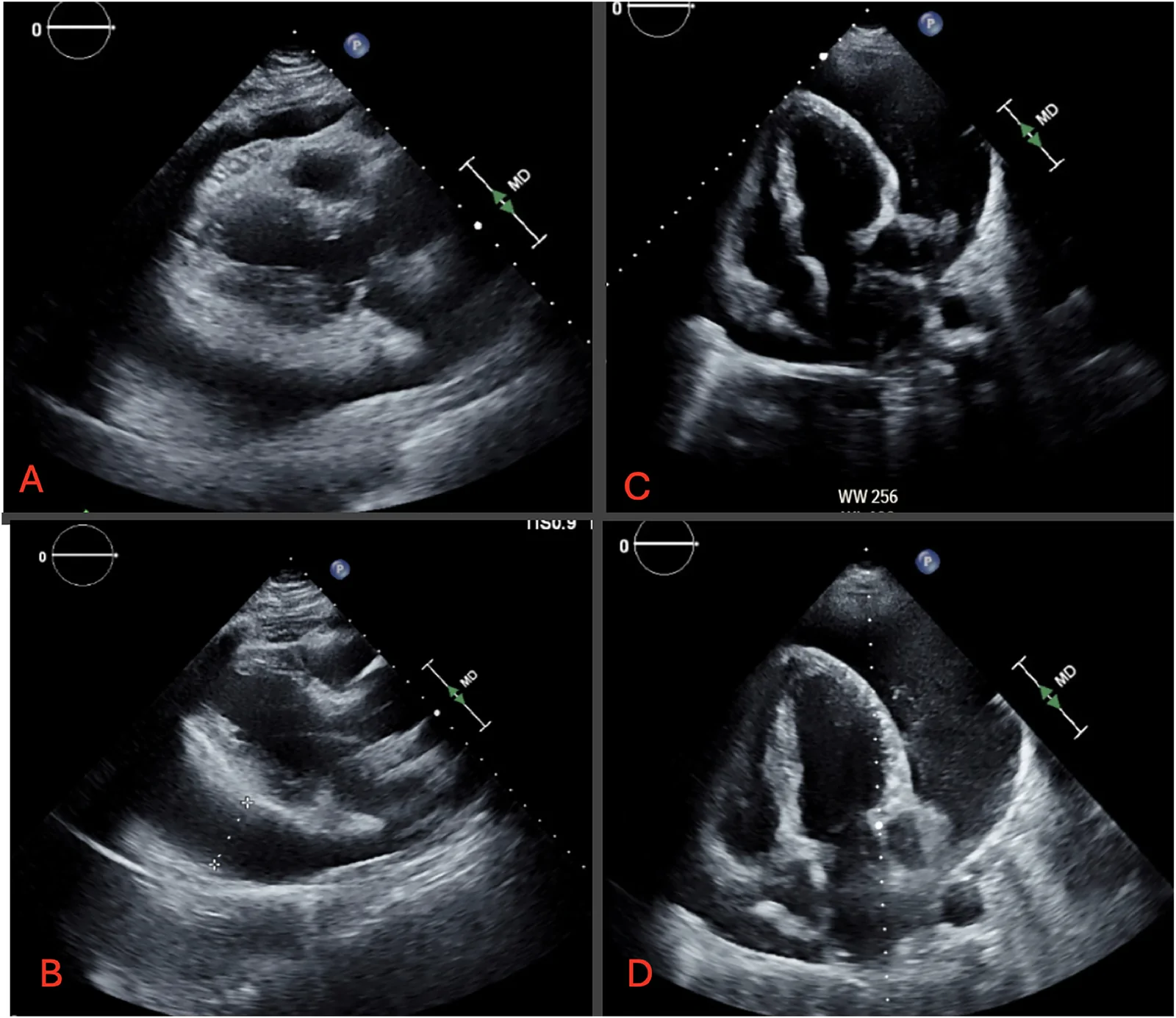
Transthoracic echocardiography: (A) and (B) parasternal long-axis (PLAX) views demonstrating a significant pericardial effusion measuring up to 5.3 cm, with evidence of cardiac tamponade, including right ventricular compression. (C) and (D) apical views again demonstrating the large pericardial effusion and findings consistent with cardiac tamponade, including right ventricular compression.

Given the extent of vascular, cardiac, and airway involvement, the tumor was deemed unresectable. Two weeks after her hospitalization, she underwent placement of a silicone Y-stent for malignant central airway obstruction, with plans to initiate palliative AIM chemotherapy—doxorubicin, ifosfamide, and mesna—one week later, with subsequent consideration of cytoreduction depending on treatment response.

One week later, when she presented for her first chemotherapy session, she was in acute hypoxemic respiratory failure with severe respiratory distress requiring emergent intubation. Bronchoscopy revealed near-total obstruction of the left mainstem bronchus and severe right-sided narrowing due to rapid tumor progression, with findings concerning for airway erosion with mediastinal tissue visible through the airway (Fig. 7). Repeat CT imaging confirmed interval tumor growth with worsening airway compromise (Fig. 8). Given the rapid disease deterioration, she was initiated on AIM chemotherapy.

**Fig. 7:**
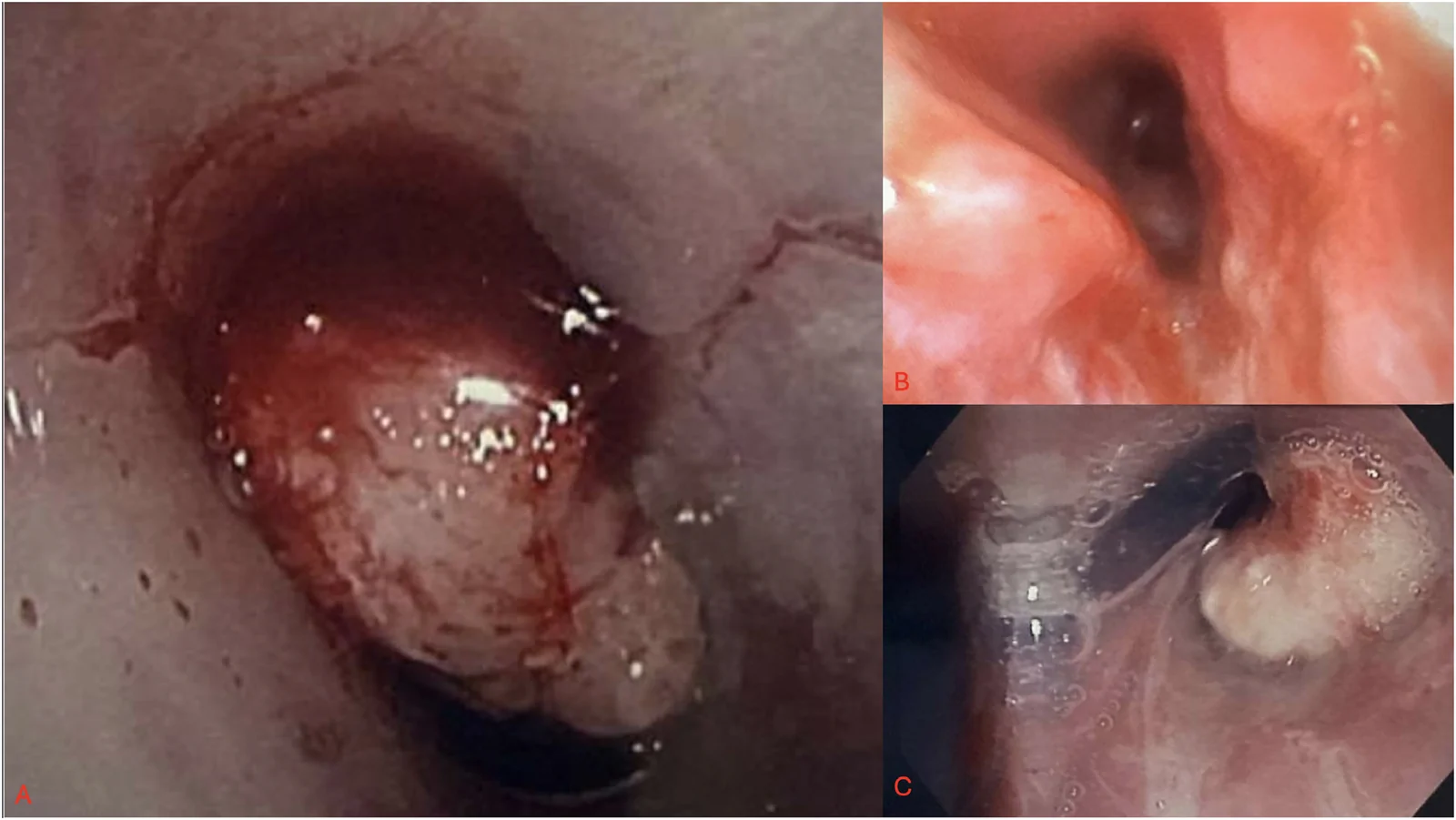
Bronchoscopy images after acute respiratory decompensation: (A) Left mainstem bronchus demonstrating a tumor causing complete obstruction. (B) The medial limb of the stent is displaced medially with partial erosion through the left mainstem bronchus. This image, obtained from within the stent, shows the medial wall of the left mainstem bronchus at the 5 o’clock position; the stent limb is partially within the area of erosion and is not coaxial with the distal airway. (C) View near the level of the carina demonstrating lateral displacement of the stent to the right, resulting in obstruction and pressure necrosis of the right mainstem bronchus.

**Fig. 8:**
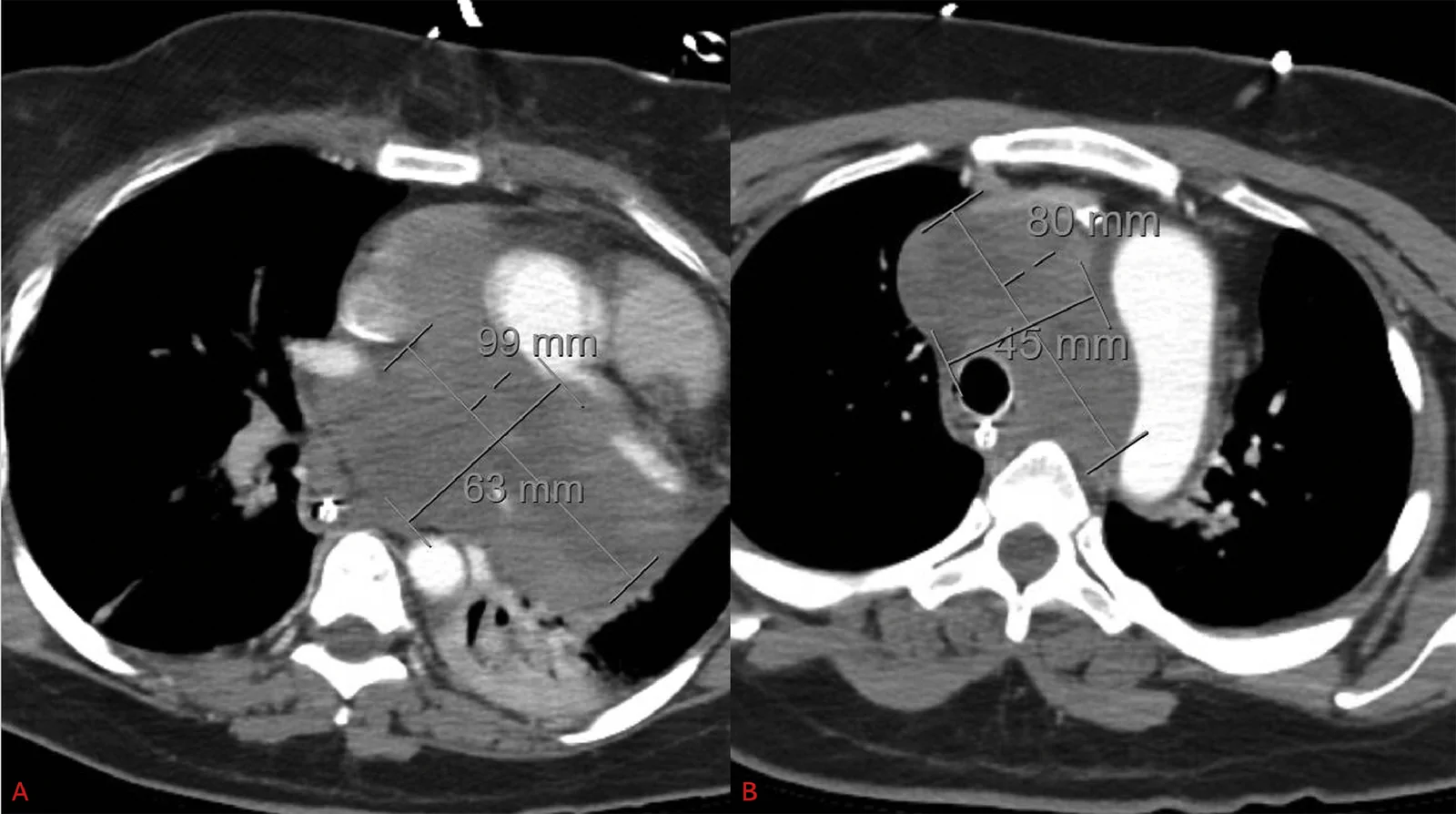
Repeat CT chest after acute respiratory decompensation: (A) and (B) demonstrate a significant interval increase in the size of the mediastinal and pericardial soft tissue mass, consistent with disease progression. (A) shows increased measurements of the posterior pericardial component, while (B) demonstrates enlargement of the anterior component.

She was subsequently transferred to a quaternary care center for advanced airway management. Despite Y-stent revision, her course was complicated by massive hemoptysis causing airway obstruction, pulseless electrical activity arrest, and death. Hemorrhage was suspected to result from tumor-related vascular invasion.

## Discussion

This case highlights the diagnostically challenging nature of primary mediastinal synovial sarcoma through three key themes: a predominantly mechanical cardiopulmonary presentation requiring emergent intervention, catastrophic hemoptysis from vascular invasion, and a rapid progression from an initially normal chest radiograph to fatal unresectable disease.

Mediastinal sarcomas often remain clinically silent until significant mass effect occurs, and advanced presentations are frequently driven by compression of adjacent structures rather than systemic tumor burden [[4], [5], [6],^8^,^9^]. Consistent with this pattern, the tumor in this case manifested primarily through progressive structural cardiopulmonary compromise rather than constitutional symptoms. The patient developed cardiac tamponade, bilateral pulmonary artery compression, left atrial compression with invasion, severe tracheobronchial obstruction, dysphagia, and orthopnea.

Among these complications, severe malignant central airway obstruction required placement of a silicone Y-stent for palliation. Airway compromise in mediastinal malignancy may result from extrinsic compression, direct airway invasion, or a combination of both. Although endobronchial stenting can provide rapid symptomatic relief and temporary stabilization, its benefit is often short-lived in aggressive infiltrative tumors, in which continued extrinsic compression or distal progression may overcome the intervention [10]. In this case, the stent functioned as a bridge rather than a definitive solution, as rapid tumor progression led to recurrent obstruction and respiratory failure.

Vascular invasion or erosion may produce catastrophic complications in patients with advanced mediastinal malignancy. In this case, massive hemoptysis was the terminal event. Although direct vascular invasion was not definitively demonstrated by imaging, bronchoscopy, or procedural evaluation, the abrupt hemorrhage in the setting of extensive mediastinal involvement raised concern for tumor-related vascular erosion and vessel rupture. Accordingly, massive hemoptysis in patients with advanced mediastinal malignancy should be recognized as a potential marker of severe local invasion and impending clinical deterioration [11,12].

The extent of local invasion also has important implications for treatment and prognosis. Synovial sarcoma is characterized by a SS18-SSX fusion gene, and prognosis is strongly dependent on complete surgical resection. However, mediastinal tumors are frequently unresectable at diagnosis because of early invasion of the heart, great vessels, and central airways [[13], [14], [15], [16]]. In a pooled analysis of 40 patients with primary mediastinal synovial sarcoma, the median overall survival was 36 months, and the 5-year overall survival rate was 35.7%. Complete surgical resection was the only factor significantly associated with improved survival, with a 5-year overall survival of 63% after complete resection compared with 0% among patients without complete resection [2]. These findings underscore the particularly poor prognosis associated with unresectable mediastinal disease.

Despite its aggressive clinical behavior, primary mediastinal synovial sarcoma remains difficult to identify radiologically. On CT, it typically appears as a large, well-circumscribed or infiltrative soft-tissue mass with heterogeneous enhancement. Areas of necrosis, hemorrhage, or cystic degeneration may contribute to internal heterogeneity, and calcification may occur, although it is not consistently present [1,17]. Imaging findings are not pathognomonic; however, a large heterogeneous mass in a younger or middle-aged adult, particularly with local compression or invasion and without features characteristic of more common mediastinal tumors, may raise suspicion for a primary mesenchymal malignancy [1,17,18]. The differential diagnosis varies according to the involved mediastinal compartment and includes thymic epithelial tumors in the anterior mediastinum, peripheral nerve sheath tumors in the posterior mediastinum, and other spindle-cell or primary mesenchymal neoplasms across compartments [1]. Magnetic resonance imaging, when available, can further characterize hemorrhagic, necrotic, and cystic components and delineate tumor relationships with adjacent mediastinal structures, assisting assessment of local extent and potential resectability [1,19]. Nevertheless, imaging remains nonspecific, and definitive diagnosis requires histopathologic, immunohistochemical, and molecular confirmation.

A central diagnostic teaching point from this case is the potential for false reassurance from an initially negative chest radiograph. The initial radiograph was interpreted as showing no mediastinal abnormality despite progressive respiratory symptoms, and retrospective review did not reveal a definite mediastinal mass or other specific radiographic abnormality. Repeat radiography six weeks later demonstrated rightward tracheal deviation, prompting cross-sectional imaging that revealed an extensive infiltrative mediastinal mass. Chest radiography has limited sensitivity for mediastinal pathology because early lesions may be subtle, may mimic benign processes, may be obscured by overlapping mediastinal and cardiopulmonary structures, or not yet produce a conspicuous contour abnormality on projectional imaging [8,20]. Thus, a normal chest radiograph should not be interpreted as excluding clinically significant mediastinal disease when symptoms persist or progress. This case underscores the importance of repeat imaging and timely escalation to cross-sectional imaging when clinical symptoms are discordant with an initially unrevealing radiograph [8].

## Conclusion

Primary mediastinal synovial sarcoma is a rare and highly aggressive malignancy that may initially present with nonspecific respiratory symptoms despite unrevealing early imaging. This case illustrates how an initially radiographically occult lesion can progress rapidly to advanced unresectable disease with life-threatening complications, including cardiac tamponade, malignant central airway obstruction, and catastrophic hemoptysis.

Careful integration of clinical progression with repeat imaging is essential, particularly when symptoms persist despite an initially negative chest radiograph [20]. Although multidisciplinary intervention may provide temporary stabilization, outcomes remain poor once extensive mediastinal invasion has occurred.

## Patient consent

Informed consent was obtained directly from the patient before her clinical decompensation, endotracheal intubation, and subsequent death for the publication of this case report, including any accompanying images. The patient was informed that all efforts would be made to protect her identity, and all identifying details have been removed to maintain confidentiality.
